# Cases of Synovial Articular Inflammation of the Knee, Treated Successfully with Urate of Ammonia*This paper was originally read before the Academy of Natural Sciences, on Tuesday December 9th, 1851, though now published by preference in a Medical Journal.

**Published:** 1852-02

**Authors:** W. E. Horner

**Affiliations:** Prof. of Anatomy in the University of Pennsylvania, Senior Surgeon of the St. Joseph’s Hospital, etc.


### MEDICAL EXAMINER,

AND

### RECORD OF MEDICAL SCIENCE.

NEW SERIES.—NO. LXXXVI.—FEBRUARY, 1852.

### ORIGINAL COMMUNICATIONS.

*Cases of Synovial Articular Inflammation of the Knee, treated successfully with Urate of Ammonia.** By W. E. HORNER, M. D., Prof. of Anatomy in the University of Pennsylvania, Senior Surgeon of the St. Joseph's Hospital, etc.

The liniment of ammonia is so well known in the treatment of chronic articular affections, that its character may be considered as settled; but my attention has been only lately called to the still higher powers of urate of ammonia, an article which, though sufficiently offensive to the olfactories, has a strong compensating quality in the efficiency of its action. My first observation in regard to it was the result of having accidentally been

* This paper was originally read before the Academy of Natural Sciences, on Tuesday December 9th, 1851, though now published by preference in a Medical Journal.

called to a poor woman who was in a state of unremitted and excruciating suffering, day and night, but especially during the latter period, from a chronic inflammation of the knee joint, attended with considerable swelling and tenderness, and some degree of redness. I made to it the ordinary applications of cold fomentations, and evaporating lotions, enjoined rest, attended to her diet and the state of her bowels, and gave opiates at night in the shape of Dover's powder. After ten or twelve days of attendance, in which no progress was made to a cure, I was much gratified on a visit to find that the pain had ceased suddenly, and that the preceding night had been spent in great ease. The strong expression of satisfaction on my part, led to a communication from her, with many apologies for herself, that, finding the disease so little abated, she had been tempted to try the remedy of a simple friend, who had been remarkably improved by it in a similar attack. This remedy was a poultice made of human urine, thickened with potter's clay, and put on as warm as one could bear it; and to be repeated when it got dry. She declared to me that this rather indelicate application had relieved her of all pain in a few hours. The fact of relief was incontestible; the question was in regard to the remedial agency of the article employed, and I therefore determined to make some experiments on the value of ammonia in combination with fine argillacious earth.

Having a similar case shortly afterwards, in the St. Joseph's Hospital, I tried in it a solution of muriate of ammonia formed into a poultice. No very distinct or satisfactory result followed, and it was discontinued. Having the idea still in my mind, and wishing to be satisfied about it, but reluctant to employ the article resorted to by the poor woman, I determined to find my urate of ammonia in some other form of an easy kind, and for that reason adopted the guano, which has so large a proportion of phosphate of lime, urea and of urate of ammonia in it. A female patient, aged 34 years, Mrs. C——, from Tamaqua in this State, who had for more than a year labored under inflammation of the right knee, was put under my charge at the St. Joseph's Hospital, October 8th, 1851. She had been well attended to by Dr. Scherner, who had conducted her through the most acute period of her complaint. The joint had suppurated, and she came to town with a small fistulous orifice on the inner side of the knee, through which a probe could be easily passed between the tibia and os femoris. From this there came daily a spoonful or more of matter, when a plug was withdrawn from the orifice. She still suffered great pain at night, the part was tender, and was in continual uneasiness, and she had some slight fever in the afternoon. Here was exactly the case to try the efficacy of urate of ammonia, as naturally formed in animals. I accordingly obtained some guano, and had it made into a hot poultice with clay. The joint was kept enveloped in the poultice with frequent changes, for nearly the remainder of the month, at the end of which time a very marked improvement had taken place in the amount of pain, and also in the degree of swelling; and the purulent discharge had almost ceased.

The application produced a very copious vesication of the knee, and it had to be weakened to reduce the caustic qualities. Having conducted this treatment as far as seemed necessary, the skin was permitted to heal. Some little pain recurring afterwards, she was blistered for it; that getting well, the emplastrum calefaciens was applied, and the leg was also kept supported by an extending band on the ankle, and a counter-extending one on the thigh, their action being sustained by a splint on the outside of the limb. At the end of six weeks, November 25th, she has left the hospital without pain or uneasiness in the knee. The joint is in a state of false anchylosis, and straight. I have covered the knee with emplastrum adhesivum, and secured it in that position with strong paste board splints, moulded to the knee; and have recommended her to keep it so for two or three months, until all danger of secondary suppuration be removed. Probably at the end of this time the judicious use of frictions and of Stromeyer's screw splint, may impart some flexion to the limb.

The Hospital record sheet shows the following details of dates, which may be inserted in this place:

*Oct.* 9. Poultice of guano, (*urate of ammonia*) and potter's clay, equal parts.

*Oct.* 10. Poultice has blistered. It was discontinued, and simple cerate applied.

*Oct.* 11. Patient has less pain; soreness of knee reduced, and not so much swelling. A poultice with one-third of the urate of ammonia, and two-thirds potter's clay.

*Oct.* 13. Vesication.

*Oct.* 14. Quantity of urate reduced to one-fourth of poultice. Treatment continued pretty much in this state to near the end of the month. Vesication by Emplast. Cantharid. about this time, but omitted on record.

*Oct.* 28. She was permitted to eat as she pleased.

*Nov.* 5. Emplastrum calefaciens.

*Nov.* 12. Discontinue emplastrum calefaciens, and re-apply the urate of ammonia as on 14th October.

While this case was in progress, another occurred in a boy who had the knee joint opened by a cut of half an inch or so in length. Synovial inflammation followed, with the ordinary symptoms. Its usual acute-period was passed through, under the depletory antiphlogistic treatment, and with evaporating lotions to the part. The disposition to fall into the chronic state, attended with tumefaction was relieved by five days use of the same argillacious, uro-ammoniacal poultice.

The Ward sheet exhibits the following entries in regard to this case: Patient, Timothy Roach, aged nineteen years, admitted September 23d, a day or two after accident. Knee painful and stiff, somewhat swollen. Rest and fomentations of warm water directed on that day. Also loss of ten ounces of blood from arm.

*Sept.* 24. Local bleeding by scarified cupping. Fomentations continued.

*Oct.* 4. Warm fomentations to this date; in the mean time an evident articular effusion has occurred into the synovial membrane of the knee. A blister plaster 4 inches by 4, was then applied.

*Oct.* 6. Blister plaster 2×3 inches.

*Oct.* 7. The patient so much relieved from pain as to be permitted to leave his bed and promenade with a crutch.

*Oct.* 9. Some aching and tumefaction indicated a persistence of articular irritation. The poultice of urate of ammonia (guano) one-fourth, potter's clay three-fourths, was then applied hot, with frequent renewals to the 14th of October, at which time all the symptoms were relieved. The patient was discharged cured on the 15th.

The above cases are reported much in outline. I shall continue, as opportunity offers, to test the value of the above remedy, and also compare its results with other remedies. It appears to me to have some special qualities, which are of a highly beneficial kind in the affections alluded to. It is so active a revulsive when applied strong, that I have no doubt of their being many cases of serous inflammation in which it may be usefully resorted to. I would here suggest a trial in puerperal peritonitis and in pleurisy. I see no objection except the odour.

The poultice of guano and clay dries very quickly, so that it is better to shield it with oiled silk or India rubber cloth. The clay I look upon as simply a vehicle, but it may also have some physiological action from its physical properties in regard to moisture.

The analysis of the best guano, by the chemist, presents the following constituents, which are mentioned here for facility of reference. The proportions will vary according to their atmospheric exposure and to the degree of adulteration in trade. As it is an expensive article for agricultural purposes, it has become common to reduce its chemical relations by the addition of common earthy substances:

Uric acid, thirty per cent.
Uric acid with ammonia.
Carbonate of ammonia.
Muriate, oxalate, and phosphate of ammonia.
Free ammonia.
Phosphate of soda.
Phosphate of lime.
Sulphate of potash and soda, and oxalate of lime.

It is the large quantity of ammonia in it which makes it so active a stimulant to vegetable growth, and so disagreeable to the smell. It, however, is not so intolerable medically, as assafetida, an article which we have but little hesitation in prescribing.

